# Selective Isolation of Surface Grain Boundaries by Oxide Dielectrics Improves Cd(Se,Te) Device Performance

**DOI:** 10.1021/acsami.4c16902

**Published:** 2025-01-24

**Authors:** B. Edward Sartor, Ryan Muzzio, Chun-Sheng Jiang, Chungho Lee, Craig L. Perkins, André D. Taylor, Matthew O. Reese

**Affiliations:** † National Renewable Energy Lab, Golden, Colorado 80401, United States; ‡ First Solar, Santa Clara, California 95050, United States; § New York University, Brooklyn, New York 11201, United States

**Keywords:** photovoltaics, CdTe, oxides, contacts, solution-processing

## Abstract

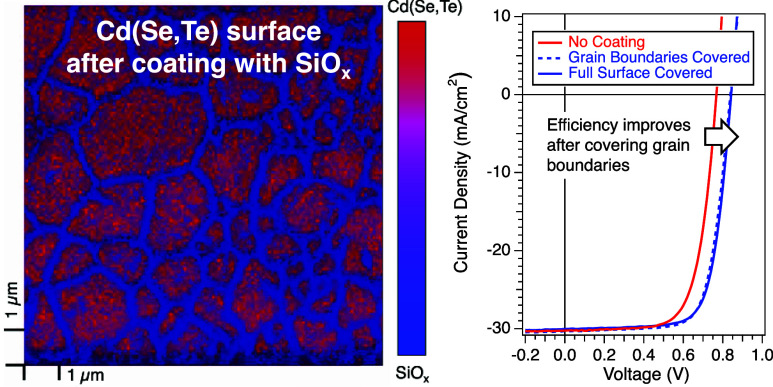

Cd(Se,Te) photovoltaics (PV) are the most widely deployed thin-film solar technology globally, yet continued efficiency improvements are stymied by challenges at the device hole contacts. The inclusion of solution-processed oxide layers such as AlGaO_*x*_ in the contact stack has yielded improved device open-circuit voltages (*V*_OC_) and fill factors (FF). However, contradictory mechanisms by which these layers improve the device properties have been proposed by the research community. We demonstrate in this work that an underappreciated property of such spin-coated layers is the preferential deposition at grain boundaries, a process that isolates the grain boundaries during contact metallization. The effects of grain-boundary isolation are probed by varying the coverage of solution-processed AlGaO_*x*_ “barrier” layers on the Cd(Se,Te) surface, quantified by scanning Auger microscopy. Examining coverage-dependent *V*_OC_ and FF, it was observed that isolating the grain boundaries during metallization is sufficient to prevent damage to the absorber that occurs in devices lacking a barrier layer, while additional coverage contributes to the increased series resistance. Such an effect is agnostic to the material used as a barrier layer, as long as the material does not itself damage the absorber. Spin-coated SiO_*x*_ was used in place of AlGaO_*x*_ for an equally beneficial effect. This grain-boundary isolation phenomenon is also observed during Mo deposition and in absorbers that have been contacted with a nitrogen-doped ZnTe layer. The mechanisms by which metallization may degrade the absorber are discussed, as are contact design strategies leveraging barrier layers, which may lead to improved device efficiencies.

## Introduction

Cd(Se,Te) photovoltaics (PV) are the most widely deployed thin-film solar technology globally and are particularly critical to the continued health of the U.S. PV market, making up ∼40% of utility-scale deployments within the U.S.^1^ Recently, several record devices have been fabricated following a reconfiguration of the dopant chemistry to accommodate group-V As and P dopants, with the most recent record reaching 23.1% photoconversion efficiency (PCE).^2−4^ However, the continued growth of the Cd(Se,Te) device PCE may be stymied by poor hole contacts in the near future, particularly when attempting to make thinner devices to enable bifaciality or alleviate concerns around Te availability.^5^ The high ionization energy of Cd(Se,Te) devices leads to the formation of detrimental Schottky barriers, which cause an accumulation of electrons at the rear contact, particularly in highly doped As-containing Cd(Se,Te) devices where transport occurs in larger quasi-neutral regions where electrons diffuse more readily. The rear surface of Cd(Se,Te) is highly defective, increasing nonradiative recombination at the back contact.^6−8^ Combined, Schottky barriers and a highly defective surface limit the hole quasi-Fermi level at the rear of the device, limiting the voltage that may be extracted from a device under operation. Current efforts to address the back contact are focused on passivating the surface to remove defects,^9,10^ introducing additional band-aligned layers to encourage electron reflection,^11,12^ or circumventing the problem by making devices thick to minimize the number of electrons that diffuse to the rear surface.

High-efficiency hole contacts often include a doped ZnTe layer followed by a metallization step, typically completed by evaporative or sputter deposition.^13,14^ Metallization is typically completed using high-work function metals such as molybdenum (4.6 eV) or gold (5.1 eV),^15^ but there are several potential concerns relating to the creation of defects during the process. Diffusion of metal interstitials has been reported to compensate for dopant chemistry. Klein et al. describe a process where metal tellurides form during deposition, releasing free cadmium or zinc interstitials, which act as double donors upon diffusion into the nominally p-type absorber, creating downward band bending and an increased back contact barrier.^16^ In the case of copper metal, the CdTe decomposition reaction to form Cu_2_Te and Cd^0^ was directly observed by Teeter using thermal desorption mass spectrometry.^17^ There are often additional layers included in the back contact architecture of high-efficiency devices, such as elemental tellurium,^18^ native oxides, or other materials, which may mediate the interaction of metallization with the Cd(Se,Te) surface.^8^ However, the role of these additional layers has not been adequately understood, even though their inclusion in device fabrication has led to improved device efficiencies.

**Figure 1:**
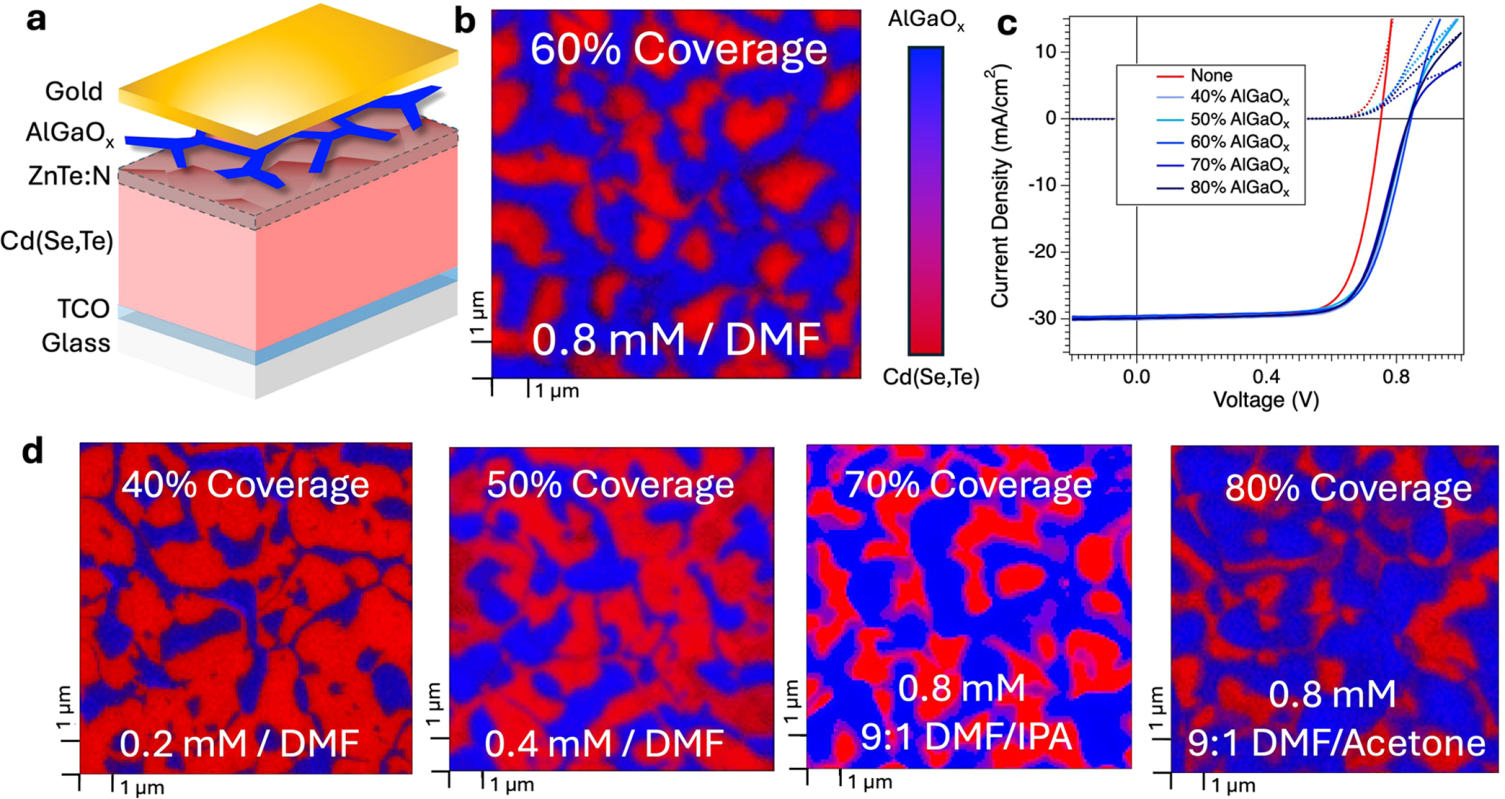
(a) Schematic of AlGaO_*x*_-contacted polycrystalline Cd(Se,Te) devices. Optional ZnTe:N buffer layer is demarcated by dashed lines. (b) Scanning Auger microscopy (SAM) map of AlGaO_*x*_ (blue) and bare Cd(Se,Te) (red) for the literature-reported method on 120 nm rms roughness Cd(Se,Te).^19^ (c) Representative *JV* data for a device without an AlGaO_*x*_ barrier layer and devices with AlGaO_*x*_ at varied coverages. (d) SAM maps of Cd(Se,Te) surfaces with varied AlGaO_*x*_ coverages from modified precursor solutions.

One such layer, AlGaO_*x*_, has been reported to passivate a defective CdTe surface in undoped devices.^19^ Recently, spin-coated AlGaO_*x*_ was used as a “barrier” layer in a bifacial contact, which improved the open-circuit voltage (*V*_OC_) of a gold metallized device from 735 to 826 mV, despite a lack of evidence for passivating behavior from the AlGaO_*x*_ layer itself as measured by time-resolved photoluminescence.^20^ Instead, a reduced back contact barrier height as measured by temperature-dependent current density–voltage measurements (*JV*(*T*)) was observed compared to a bare surface metallized by gold. By examination of the contact area with scanning auger microscopy (SAM) prior to metallization, it was observed that the coverage of AlGaO_*x*_ on the surface was highly inhomogeneous (Figure 1b). Discontinuous patches of bare Cd(Se,Te) were observed in a continuous AlGaO_*x*_ film (Figure 1a). Furthermore, reducing the concentration of nitrate salt precursors used to form the AlGaO_*x*_ barrier layer lowered the final surface coverage, and mixing more nonpolar solvents into the dimethylformamide (DMF) solvent modified the wetting behavior of the precursor solution during spin coating, resulting in higher coverage. The ability to control the surface coverage of the AlGaO_*x*_ film yielded a tool to probe the effect of inhomogeneous barrier layers during the metallization of Cd(Se,Te).

In this work, we demonstrate that solution-processed barrier layers can preferentially deposit at grain boundaries and prevent detrimental interactions with the Cd(Se,Te) absorber during metallization. Isolation of grain boundaries during metallization is crucial to prevent degradation of the Cd(Se,Te) absorber, but the effect of additional surface coverage by AlGaO_*x*_ barrier layers is minimal. By substituting chemically inert SiO_*x*_ for AlGaO_*x*_, the latter of which is produced via chemical decomposition of nitrate salts,^21^ we demonstrate that the grain-boundary isolation effect is not unique to the identity of the material used to cover the grain boundaries. The presence of this effect is demonstrated with ZnTe:N transport layers as well as during metallization with other metals beyond gold. The possible mechanisms of absorber degradation are discussed.

## Results and Discussion

SAM was used to examine Cd(Se,Te) absorbers, which had been treated with AlGaO_*x*_ (Figure 1b). CdTe regions are shown as red in the maps and are overlaid with oxygen map data shown in blue, with oxygen being a good proxy for AlGaOx. (Figure S1). Using 0.8 mM AlGaO_*x*_ in DMF precursor solution during spin coating, approximately 60% of the surface is covered with the AlGaO_*x*_ barrier layer after annealing, leaving isolated bare Cd(Se,Te) regions exposed. By reducing the concentration to 0.6 and 0.4 mM, the surface coverage of AlGaO_*x*_ was modified to 50 and 40%, respectively. Changing the wetting behavior of the precursor solution by introducing less polar solvents also resulted in a modified surface coverage, with 10% IPA:DMF and 10% Acetone:DMF increasing surface coverage to 70 and 80%. The facile modification of surface coverage (Figure 1d) by changing the concentration or solvent makeup yields a convenient system for probing the effects of inhomogeneous barrier layers. Even at high coverages, pinholes were observed, preventing 100% coverage of the surface by the oxide layer. Distinctive “grain-like” features appear in the AES image of the 0.4 mM sample, where the AlGaO_*x*_ layer appears to colocate with grain boundaries. Because SEM and AES images are only indirect indicators of grain boundaries, in Figure 3, we utilize electron-backscatter diffraction (EBSD) to confirm that our oxide buffer layers indeed preferentially deposit on grain boundaries.

**Figure 2:**
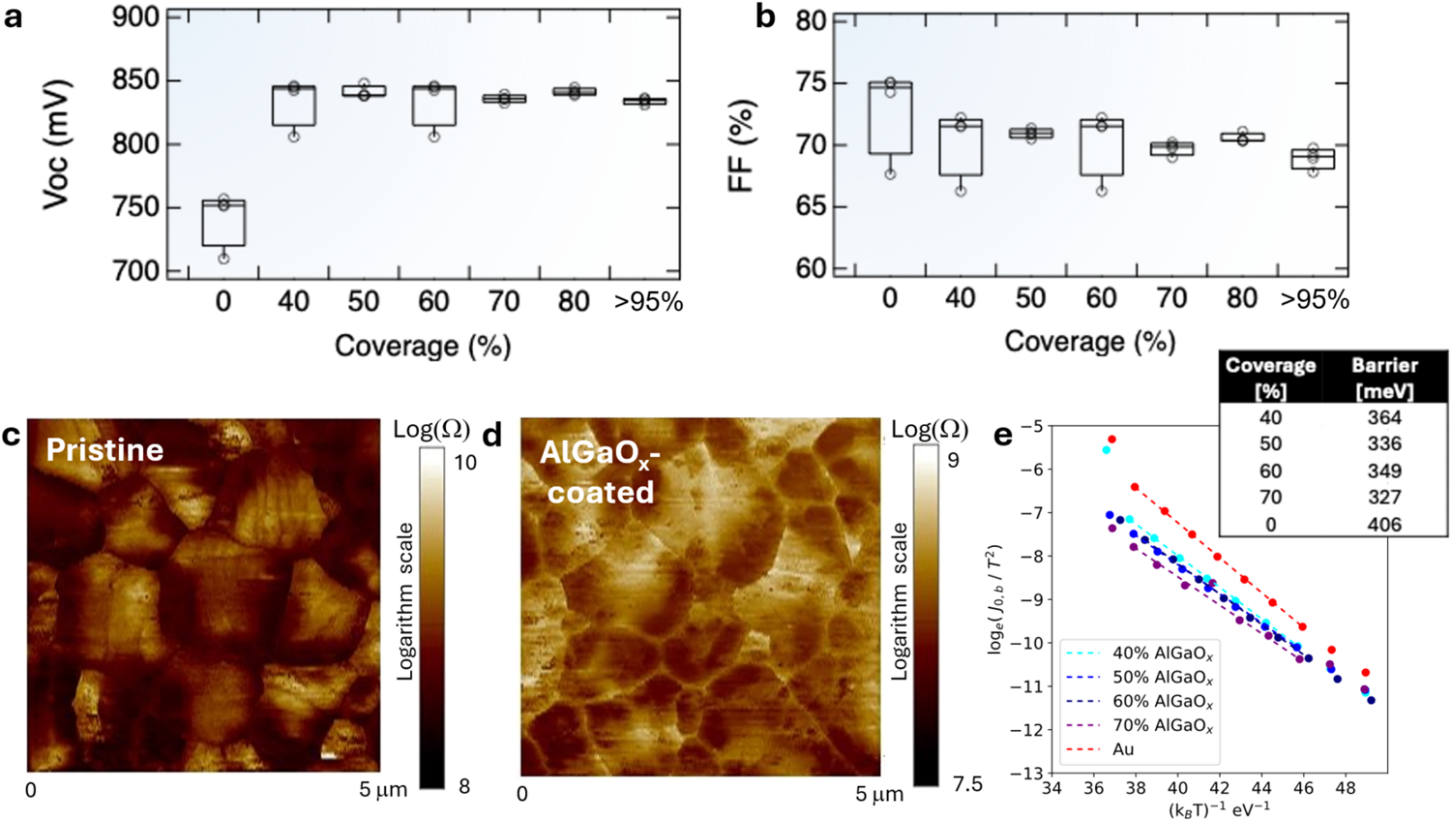
Box plots of the (a) *V*_OC_ and (b) fill factor (FF) for devices with AlGaO_*x*_ coverage varied between 0% and near 100%. Scanning-spreading resistance microscopy (SSRM) imaging of Cd(Se,Te) devices prior to metallization (c) without a barrier layer and (d) with 60% coverage by AlGaO_*x*_. The resistance of the grain boundaries relative to the grain interiors is inverted by the inclusion of a barrier layer. (e) Arrhenius plots constructed from *JV*(*T*) measurements of devices with AlGaO_*x*_ coverage varied between 0 and 80%. The inset table tracks barrier heights extracted from the slope.

Analysis of the current density–voltage (*JV*) measurements (Figure 1c) shows a remarkably similar response between AGO-coated samples of different coverages, each of which outperforms samples in which bare Cd(Se,Te) is directly metallized. Examination of parameters extracted from *JV* measurements across sample sets shows that *V*_OC_ does not trend with surface coverage after the lowest surface coverage tested (40% coverage by AlGaO_*x*_), with the treated samples yielding voltages between 840 and 850 mV (Figure 2a). Gold-only samples (0% coverage) have a depressed *V*_OC_ of only 750 mV. Fill factor (FF), however, seems to trend toward lower values for higher coverages by AlGaO_*x*_ (Figure 2b). A “roll-over” effect can be clearly seen at forward bias in samples treated with AlGaO_*x*_, indicating an electronic barrier at the back contact.^22^ This effect induces a reduction in FF due to the voltage-dependent resistance. Short-circuit current (*J*_SC_) was consistently 29–30 mA/cm^2^. In the case of inhomogeneous contact barrier heights at the rear surface, current will transport more easily across regions with lower contact barriers.^23^ The trend of decreasing FF with increasing surface coverages suggests that more AlGaO_*x*_ leads to higher series resistance or increased barriers in the regions that are coated. The maximum benefit of the AlGaO_*x*_ barrier layer is observed at lower coverages.

Scanning-spreading resistance microscopy (SSRM) is an atomic force microscopy-based scanning-probe technique for mapping local nm-scale electronic resistivity by applying a bias voltage between a conductive probe and the front contact TCO and measuring the current flowing through the probe.^24,25^ The measured total resistance is dominated by the sample’s resistance in a hemisphere of <50 nm beneath the probe. The probe/sample contact resistance is minimized much smaller than sample’s spreading resistance by applying a large forward probe/sample bias voltage (>5 V) and a large contact force in ∼ mN. SSRM was conducted on both pristine and AlGaO_*x*_-coated absorbers at forward bias, showing that the rear surface of a pristine Cd(Se,Te) absorber has grain interiors with a higher hole resistance compared to that of grain boundaries (Figure 2c). When the samples were coated with AlGaO_*x*_, the relationship between grain interior and grain-boundary resistance was inverted, suggesting that the most favorable pathway for hole transport shifted from grain boundaries to grain interiors (Figure 2d). Because of its high bandgap, generally low conductivity, and decreasing FF with increasing coverage, it is suspected that AlGaO_*x*_ is acting as an insulator rather than contributing to any modification of p-type carrier density at the grain boundary, explaining the increased local resistance.

*JV*(*T*) measurements do not yield a consistent trend between different coverages by AlGaO_*x*_. Contact barrier heights (Φ_B_) as extracted from the Arrhenius relation of back contact saturation current *J*_0,*b*_ and temperature show that barrier heights for AlGaO_*x*_-coated samples are between 320 and 370 mV. In comparison, gold-only devices have a higher contact barrier of 410 mV, consistent with previous investigations. In the case of inhomogeneous Schottky barriers, extraction of an average Φ_B_ from *JV*(*T*) measurements is known to preferentially sample regions of lower contact barrier heights.

Examining SSRM and *JV*(*T*) measurements, it is apparent that the largest impact of AlGaO_*x*_ deposition on device performance occurs at grain boundaries. At the lowest tested coverage of AlGaO_*x*_, 40%, it is expected that the coverage is mostly occurring at the grain boundaries, which isolates them from the metallization process. However, this leaves the role of AlGaO_*x*_ uncertain. While previously thought of as a means of controlling band alignment at the rear interface,^19,20^ the insensitivity of *JV*(*T*) to surface coverage and high resistance at AlGaO_*x*_-coated grain boundaries as measured by SSRM suggest that AlGaO_*x*_ does not improve contact barrier heights. Rather than acting as a low-barrier contact, AlGaO_*x*_ is most beneficial for its grain-boundary isolation effect. Such an effect should be agnostic to the material used, and thus, a generic oxide such as SiO_*x*_ would also be expected to perform in the same capacity. An unreactive oxide grown at ambient temperature may be preferred if there is potential for a detrimental interaction between the oxide film metals and the Cd(Se,Te) absorber or thermal degradation of the film stack.

**Figure 3:**
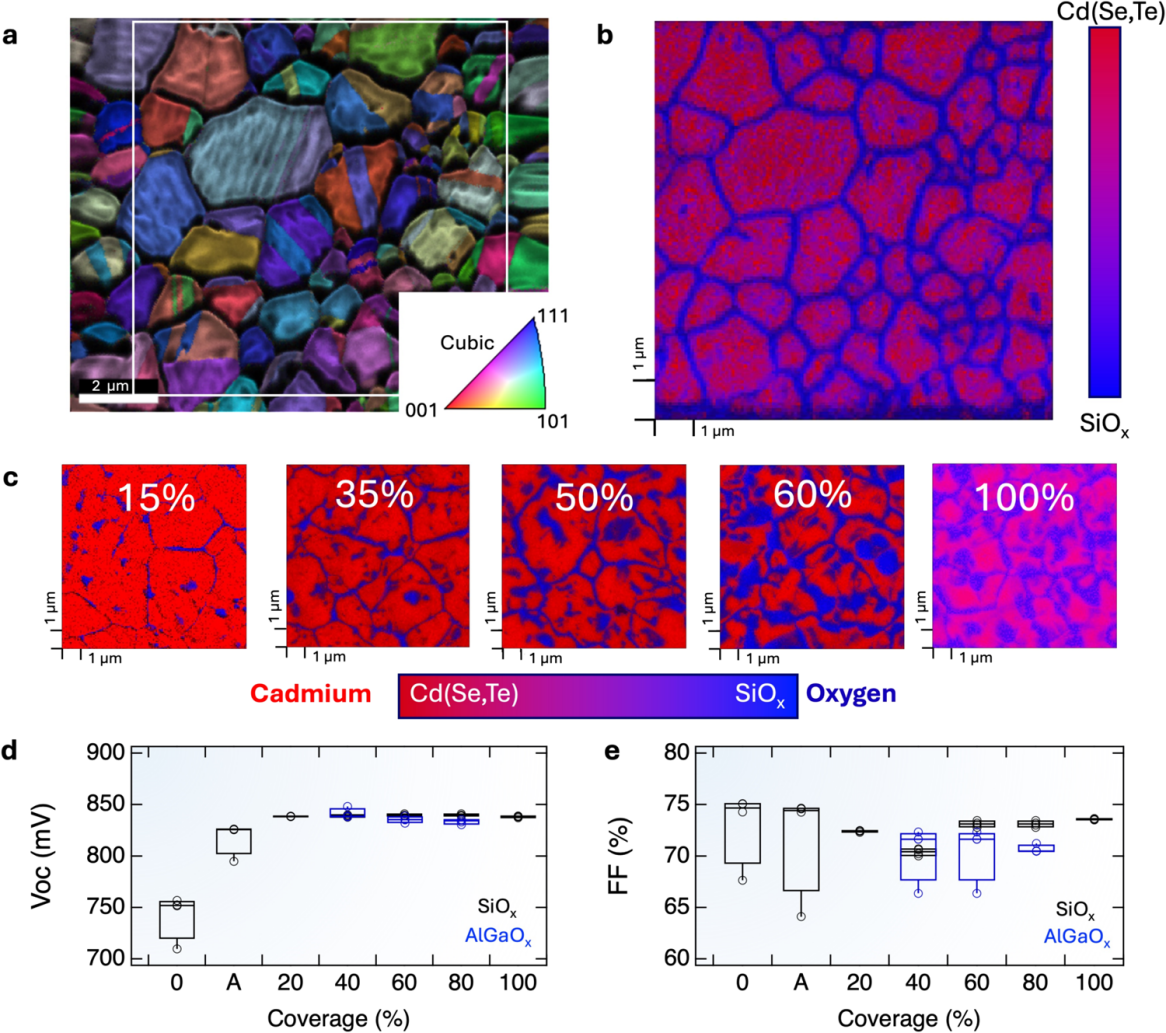
(a) Electron backscatter diffraction image of a polished Cd(Se,Te) surface coated with SiO_*x*_ showing grain orientations and the (b) SAM image of the same region depicting the presence of SiO_*x*_ in the grain boundaries. (c) SAM images of the as-deposited Cd(Se,Te) surfaces with varied coverage of SiO_*x*_ show increased grain-boundary selectivity and similarly tunable surface coverage to AlGaO_*x*_. Box plots of the (d) *V*_OC_ and (e) FF for devices with SiO_*x*_ coverage varied between 0 and 100%. “*A*” corresponds to a sample that has been annealed in air at 220 °C but not coated with a barrier layer.

SiO_*x*_ was deposited on Cd(Se,Te) absorbers from commercially available sol–gel precursors in DMF and annealed under the same conditions as AlGaO_*x*_, providing a one-to-one comparison. In one polished Cd(Se,Te) sample, colocated EBSD (Figure 3a) and AES (Figure 3b) show that SiO_*x*_ preferentially deposits at grain boundaries. In both polished and unpolished Cd(Se,Te) samples, grain boundaries produce valleys in the surface, such that the mechanism of grain-boundary deposition selectivity is likely the same and driven by the topography of the sample. All other samples investigated were unpolished. AES images show a similar tunability of surface coverage for SiO_*x*_ on unpolished samples as was observed for AlGaO_*x*_, with a very distinct grain-boundary isolation effect observable at 15% surface coverage.

*JV* parameter analysis yields a very tight distribution for *V*_OC_ in all SiO_*x*_-treated devices, from 20% surface coverage to 100% surface coverage, with the *V*_OC_ for all samples between 836 and 842 mV. FF is consistently above 70% for all surface coverages except 40%, even slightly increasing at 100% surface coverage. The additional thickness of SiO_*x*_ achieved by increasing the concentration increases series resistance and leads to a reduced FF. A control sample was annealed in air at 220 °C following the same annealing conditions as AlGaO_*x*_ and SiO_*x*_-coated films, and the annealing process improves the *V*_OC_ somewhat, but not to the extent that it is improved by adding SiO_*x*_ or AlGaO_*x*_. However, the FF is slightly higher for uncoated samples.

**Figure 4:**
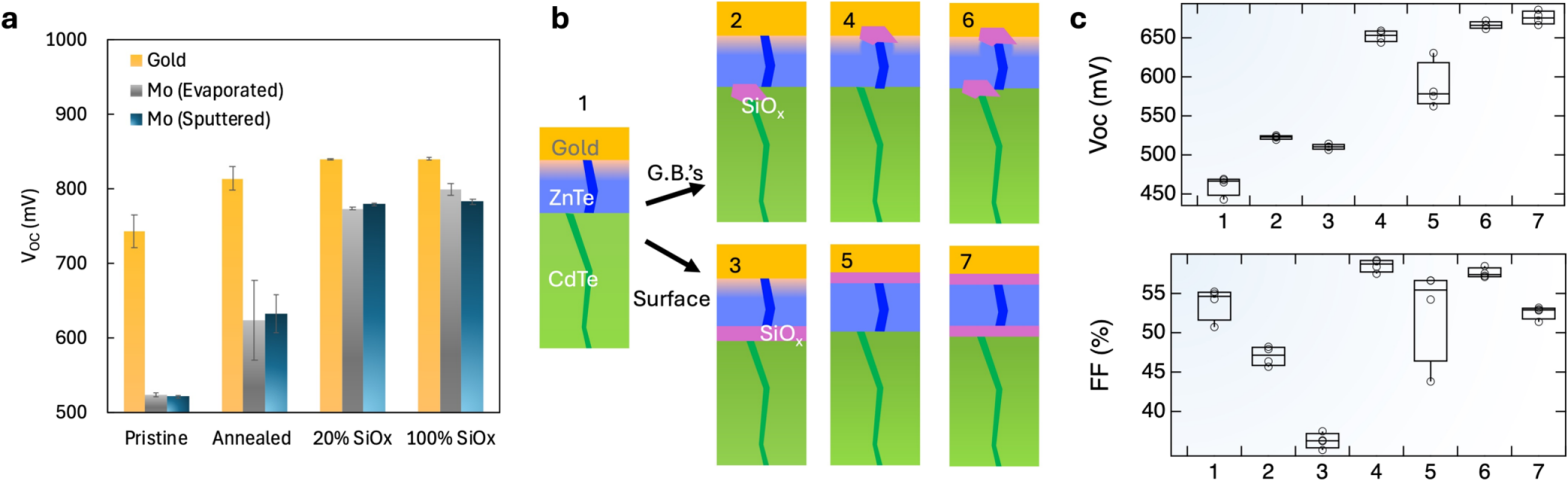
(a) *V*_OC_ comparisons of 8 devices for gold and molybdenum metallization. Molybdenum was deposited by evaporation and sputtering, with little difference observed between the deposition methods. (b) Device structures for incorporating SiO_*x*_ barrier layers before and/or after the ZnTe:N layer. SiO_*x*_ is applied to have either 20 or 100% surface coverage, resulting in the coverage of grain boundaries (“G.B.’s”: devices 2, 4, 6) or the entire surface (devices 3, 5, 7). The interfaces in the Cd(Se,Te)/ZnTe:N/Au structure are selectively SiO_*x*_ treated at the Cd(Se,Te)/ZnTe:N interface (2,3); the ZnTe:N/Au interface (4, 5); or both interfaces (6, 7). (c) Device parameters extracted from *JV* measurements, numbers along the *x*-axis correspond to structures illustrated in (b).

Both spin-coated SiO_*x*_ and AlGaO_*x*_ effectively isolate grain boundaries prior to metallization, even at low surface coverages. Grain-boundary isolation dramatically improves device *V*_OC_ and maintains a good FF when surface coverage is concentrated at the grain boundaries. This effect is observed when metallizing with gold but is also observed in devices metallized with evaporated or sputtered molybdenum (Figure 4a). This effect foreseeably occurs with most evaporated or sputtered metals that have a propensity to form stable tellurides.

Grain-boundary isolation also imparts a benefit when metalizing together with a ZnTe:N buffer layer. While ZnTe:N has been shown to improve CdTe hole contacts, device performances are lower due to a poorly tuned ZnTe:N deposition and could be reasonably expected to improve with optimization. A series of devices were fabricated to test the impact of SiO_*x*_ barrier layer incorporation coupled with ZnTe:N deposition, where the SiO_*x*_ was applied to the Cd(Se,Te)/ZnTe interface, the ZnTe/Au interface, or both. The effect of SiO_*x*_ was evaluated at two different coverages: 20% coverage SiO_*x*_, which isolated grain boundaries but left exposed semiconductor, and 100% coverage SiO_*x*_, which blankets the entire surface. The control devices with a sputtered ZnTe:N/Au contact had a low *V*_OC_ of only 460 mV. Applying SiO_*x*_ at the Cd(Se,Te)/ZnTe:N interface yielded a slight improvement in *V*_OC_ to 520 mV but imparted a reduction in device FF. Notably, the FF reduction was greater in the device with 100% surface coverage, while the *V*_OC_ improvement was similar. The most dramatic *V*_OC_ improvement was observed in devices with a SiO_*x*_ barrier layer applied at the ZnTe:N/Au interface, with grain-boundary isolation outperforming 100% surface coverage. Applying SiO_*x*_ at both the Cd(Se,Te)/ZnTe:N and the ZnTe:N/Au interfaces resulted in a slight improvement in *V*_OC_, particularly for the sample with entirely coated surfaces, but introduces additional series resistance, which decreases FF. The results with ZnTe:N demonstrate that grain-boundary isolation is more critical for the grain boundaries that are being directly metallized but that there may still be some damage occurring at the Cd(Se,Te)/ZnTe:N during the ZnTe:N sputtering process.

There are several mechanisms by which grain-boundary protection during metallization may yield improved device performance. Grain boundaries provide pathways for compensating cations to rapidly diffuse into the bulk of the absorber, even to the front of the device, where they may impact the fields of the main junction or induce high rates of recombination in the bulk. Compensating metals such as Au and Mo may also rapidly diffuse through devices via the grain boundaries,^26^ as can free cadmium interstitials generated by the formation of metal tellurides AuTe or MoTe at the rear surface.^27,28^ Impeding the access of these species to grain boundaries may limit their ingress into the device. Grain boundaries are also typically more reactive than crystalline surfaces due to a higher concentration of extended defects and dangling bonds. Reactive grain boundaries may be more likely to produce free cadmium than crystalline surfaces; thus, covering the grain boundaries during metallization reduces the concentration of free cadmium in devices. Unintentional selective area deposition on grain boundaries by other solution-coated materials could contribute to grain-boundary isolation and improve device performance. The exact mechanism by which our spin-coating process achieves selective area deposition on grain boundaries on the back surface of CdTe is not understood at this point but is the focus of an ongoing investigation. Possible mechanisms for the localization of our oxide buffer layers include capillary action and hydrophilic interactions between grain-boundary species and the precursors.

## Conclusions

Solution-processed AlGaO_*x*_ and SiO_*x*_ barrier layers can preferentially deposit at grain boundaries and prevent detrimental interactions of reactive metal species with the Cd(Se,Te) absorber during metallization. The identity of the spin-coated oxide does not impact the benefit imparted by the grain-boundary isolation behavior, but some oxides may have other beneficial or detrimental interactions with the absorber or interface. Grain-boundary isolation during metallization is crucial to preventing degradation of the Cd(Se,Te) absorber, but the effect of additional surface coverage by oxide barrier layers is minimal. Higher surface coverages can impart increased series resistances, which diminish device FF. Isolation of grain boundaries during application and metallization of ZnTe:N contacts is demonstrated to improve device *V*_OC_. Absorber degradation during metallization with Mo is also observed and is expected to occur in the presence of other evaporated or sputtered metals. However, if the degradation mechanism is due to the liberation of free cadmium due to the formation of metal tellurides during metallization, contact materials that do not form metal tellurides may be beneficial to avoid this effect entirely. Such contacts could include elemental Te, TeO_2_, carbon, or Van-der-Waal materials such as MXenes.^18,19,29^ The identification of a refractory material that forms a passivated ohmic hole contact with the interior of Cd(Se,Te) grains still remains a pathway to continued improvements in PCE and tellurium intensity per Watt. Identification of the species and mechanism responsible for the degradation of absorbers during grain-boundary metallization would be very helpful in aiding future back contact design.

## Experimental Section

### Sample Preparation

Partially completed Arsenic-doped Cd(Se)Te devices were provided by First Solar. Al(NO_3_)_3_·9H_2_O (CAS: 7784-27-2), Ga(NO_3_)_3_·*x*H_2_O, *N*,*N*-dimethylformamide (DMF), isopropanol, and acetone were purchased from Millipore Sigma. SiO_*x*_ precursor NDG-7000R was purchased from Desert Glass. AlGaO_*x*_ precursor solutions were formulated by reaching the specified molarity of a 5:3 Al:Ga precursor using Al(NO_3_)_3_·9H_2_O and of Ga(NO_3_)_3_·*x*H_2_O in DMF. SiO_*x*_ solutions were formulated by adding a % v/v of NDG-7000R to DMF, from 1 to 5%.

First Solar absorbers were washed with DI water prior to oxide deposition. AlGaO_*x*_ or SiO_*x*_ was deposited via spin coating at 1000 rpm for 10 s and 2500 rpm for 50 s, followed by a 220 °C hot plate anneal for 20 min in ambient air. The devices were then loaded into an Angstrom thermal evaporation system, where 100 nm of 7N gold was thermally evaporated onto the surface. 50 nm of ZnTe:N was deposited via reactive sputtering in the relevant samples.

### Analysis Techniques

*JV* and *JV*(*T*) were measured on instruments designed and built at NREL. SAM, SEM, and EBSD were performed using a Physical Electronics 710 Scanning Auger Nanoprobe with a 10 keV and 10 nA electron beam, all at room temperature. SSRM is based on the contact mode of atomic force microscopy using a Bruker Dimension Icon with a Nanoscope V controller. A logarithmic amplifier with a wide resistance range of 10^3^–10^14^ W was used, enabled by a Bruker SSRM module. The probe is a highly doped diamond-coated Si tip with a stiff cantilever spring constant of 80 N/m (Bruker DDESP-V2) to enhance the probe/sample contact force.

## Supporting Information Available

Scanning Auger emission correlations between Si/Al and O; surface profilometry data, *JV* data for ZnTe:N devices (PDF)
