# Supplementary material for: Selective Isolation of Surface Grain Boundaries by Oxide Dielectrics Improves Cd(Se,Te) Device Performance
Source: ACS Appl Mater Interfaces. 2025 Jan 24;17(5):7641–7. doi: 10.1021/acsami.4c16902 (PMC11803562; doi:10.1021/acsami.4c16902)
Supplement: Supplementary file 1 — am4c16902_si_001.pdf [file am4c16902_si_001.pdf]

Supporting Information

Selective isolation of surface grain boundaries by oxide dielectrics improves Cd(Se,Te) device performance

B. Edward Sartor,[1] Ryan Muzzio,[1] Chun-Sheng Jiang,[1] Chungho Lee,[2] Craig L. Perkins,[1] André D. Taylor,[3] Matthew O. Reese[1]*

[1]National Renewable Energy Lab, Golden, Colorado, 80401, USA

[2]First Solar, Santa Clara, California, 95050, USA

[3]New York University, Brooklyn, New York, 11201, USA

Corresponding author contact info:

ed.sartor@nrel.gov; 15013 Denver West Parkway, Golden; 80403 CO

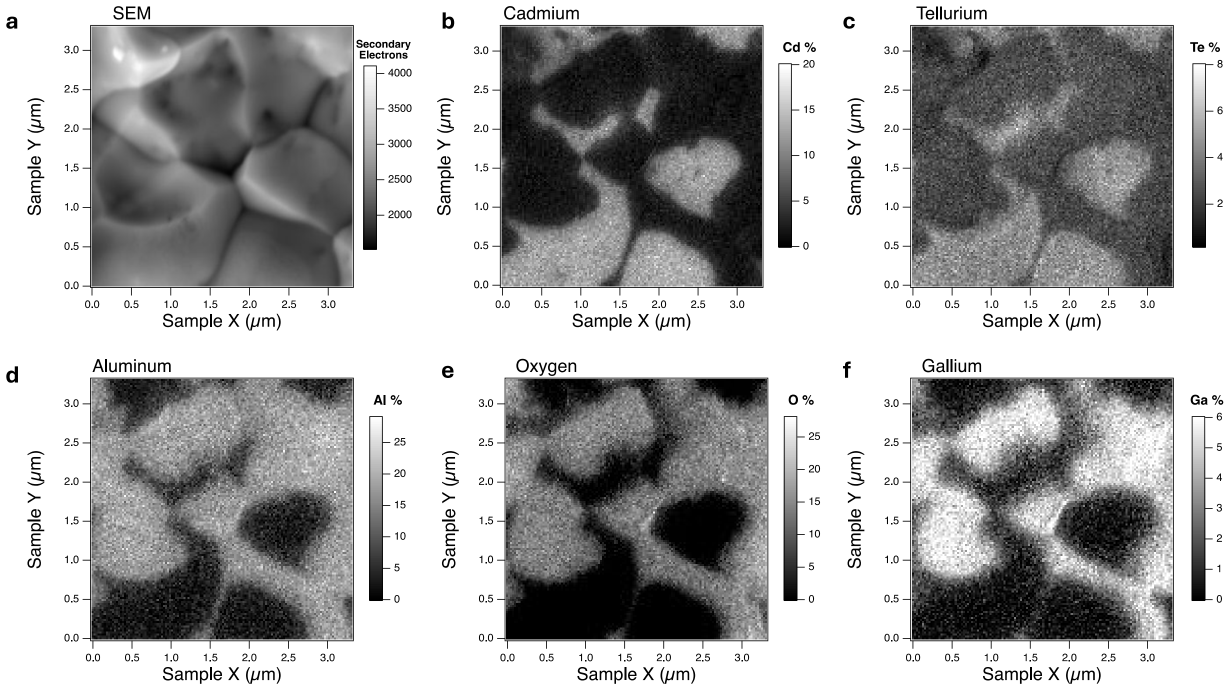

Figure S1. a) Scanning electron microscopy (SEM) image of surface topography. The polycrystalline nature of this surface is evident from the image, but individual grains cannot be identified from this image. b,c) Scanning Auger microscopy (SAM) image of cadmium and tellurium respectively on a $AlGaO_x$ coated Cd(Se,Te) surface. Lighter patches indicate a bare Cd(Se,Te) surface. d,e,f) Correlated SAM images of aluminum, oxygen, and gallium respectively. The high degree of correlation between the aluminum, gallium, and oxygen images, and the anti-correlation of oxygen with cadmium and tellurium that oxygen, indicate that oxygen is a reasonable proxy for gallium and aluminum, indicating the presence of the $AlGaO_x$ oxide at the regions where high oxygen signal is observed. This facilitates easier data collection due to the high sensitivity of the SAM measurement to oxygen because oxygen is the primary element in the oxides.[1]

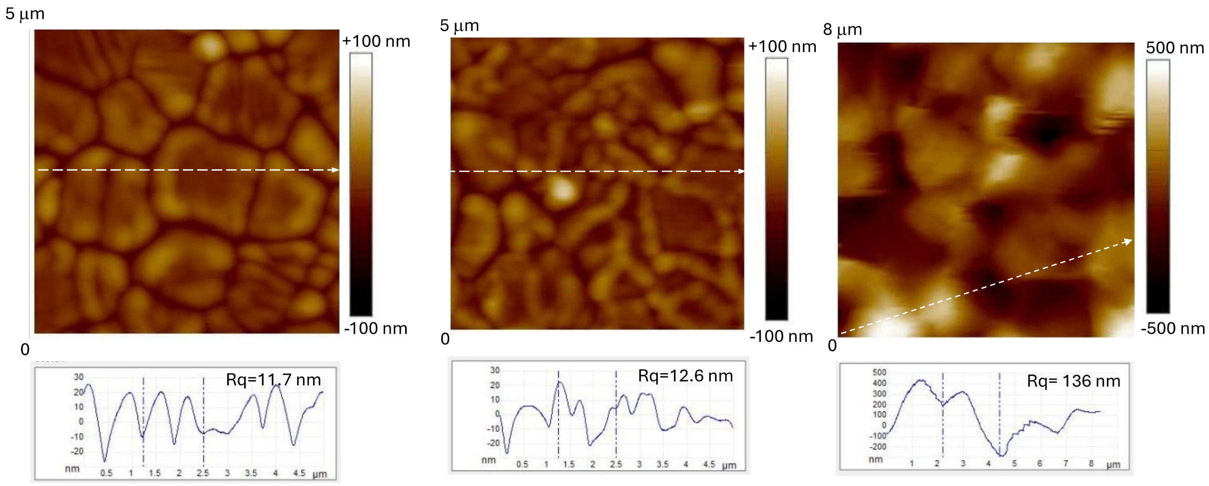

Figure S2. Profilometry data for a polished First Solar substrate (left), a polished First Solar substrate coated with SiO_x (center), and an "as-received First Solar substrate. Polishing reduced the RMS surface roughness from 136 nm to 11.7 nm.

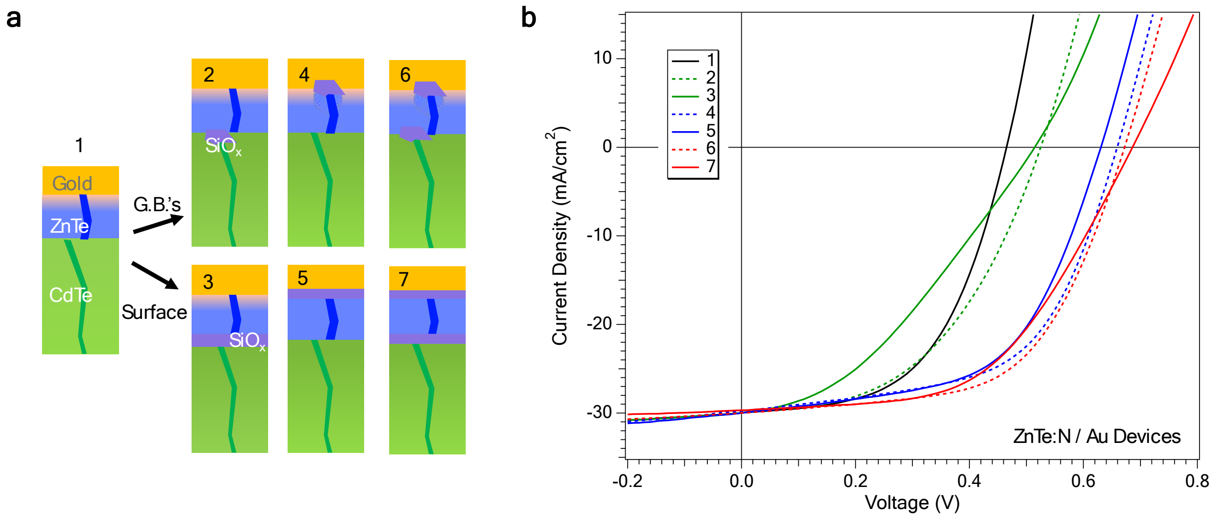

Figure S3. a) Device structures for incorporating SiO_x barrier layers before and/or after the ZnTe:N layer. SiO_x is applied to have either 20% or 100% surface coverage, resulting in coverage of grain boundaries ("G.B.'s": devices 2,4,6) or the entire surface (devices 3,5,7). The interfaces in the Cd(Se,Te)/ZnTe:N/Au structure are selectively SiO_x treated at the Cd(Se,Te) / ZnTe:N interface (2,3); the ZnTe:N / Au interface (4,5); or both interfaces (6,7) b) *JV* curves for each device structure.
